# A Brief mHealth-Based Psychological Intervention in Emotion Regulation to Promote Positive Subjective Well-Being in Cardiovascular Disease Patients: A Non-Randomized Controlled Trial

**DOI:** 10.3390/healthcare10091640

**Published:** 2022-08-28

**Authors:** Naima Z. Farhane-Medina, Rosario Castillo-Mayén, Bárbara Luque, Sebastián J. Rubio, Tamara Gutiérrez-Domingo, Esther Cuadrado, Alicia Arenas, Carmen Tabernero

**Affiliations:** 1 Maimonides Biomedical Research Institute of Cordoba (IMIBIC), 14071 Córdoba, Spain; 2 Department of Psychology, University of Cordoba, 14071 Córdoba, Spain; 3 Department of Didactics of Experimental Sciences, University of Cordoba, 14071 Córdoba, Spain; 4 Department of Psychology, University of Seville, 41018 Seville, Spain; 5 Institute of Neurosciences of Castilla y León (INCYL), University of Salamanca, 37007 Salamanca, Spain

**Keywords:** cardiovascular disease, positive subjective well-being, emotion regulation, brief psychological intervention, mHealth

## Abstract

The emotional impact that a cardiovascular disease may have on a person’s life can affect the prognosis and comorbidity of the disease. Therefore, emotion regulation is most important for the management of the disease. The aim of this study was to analyze the effectiveness of a brief mHealth psychological intervention in emotion regulation to promote positive subjective well-being in cardiovascular disease patients. The study sample (*N* = 69, 63.7 ± 11.5 years) was allocated to either the experimental group (*n* = 34) or control group (*n* = 35). The intervention consisted of a psychoeducational session in emotion regulation and an mHealth-based intervention for 2 weeks. Positive subjective well-being as a primary outcome and self-efficacy to manage the disease as a secondary outcome were assessed at five time points evaluated over a period of 6 weeks. The experimental group showed higher improvement in positive subjective well-being and self-efficacy for managing the disease compared to the control group over time. The experimental group also improved after the intervention on all outcome measures. Brief mHealth interventions in emotion regulation might be effective for improving positive subjective well-being and self-efficacy to manage the disease in cardiovascular patients.

## 1. Introduction

The prevalence of cardiovascular disease (CVD) seems to be stable over time, being the first cause of death and a major loss of health worldwide [1,2]. Empiric evidence has proven that the risk of developing CVD comes not only from biological factors but also from behavioral, psychological, and social factors, which, according to a biopsychosocial model of health, interact with each other [3]. In the same way, the consequences or repercussions of CVD involve the daily life of the people who suffer it, their quality of life, and the emotional balance to cope with it [4]. Therefore, depression [5], anxiety, and stress [6] may appear after CVD. This could be a result of coping with the chronic disease in itself, as well as a consequence of the multi-level changes that these patients have to face after the diagnosis. Comorbid anxiety-depressive symptomatology may complicate their recovery [7] and can also affect their self-efficacy for managing the disease, resulting in the abandonment of medical recommendations, putting their health at risk. Consequently, psychological interventions are needed in order to help patients regulate these emotions in a healthy manner to prevent comorbidity and promote a healthy quality of life. Thus, the purpose of this study is to develop and test a brief mHealth-based psychological intervention in emotion regulation to promote positive subjective well-being and self-efficacy for managing the disease in CVD patients.

### 1.1. Brief Psychological Interventions

There are already studies that have incorporated brief psychological interventions into cardiac rehabilitation [8,9]. Their low cost and promising results that seem to have lasting benefits [10,11,12] place this type of intervention as an interesting supplement to be considered when treating patients with CVD [13]. The chronic nature of this disease implies the need to adopt healthy habits on a continuous basis. The difficulty of modifying lifestyle and adherence to treatment is added to the anxiety-depressive symptomatology as possible conditioning factors for the physical vulnerability of a cardiac pathology [14,15]. Brief psychological interventions improve the prognosis of cardiac rehabilitation, helping patients to adapt to the long-term challenges related to CVD [8]. This kind of intervention appears to have a positive effect in this sense, enhancing psychological well-being, reducing anxiety and depressive symptoms, encouraging the promotion of healthy habits, promoting awareness of the disease, and controlling risk factors [8,9].

### 1.2. Emotion Regulation and Positive Well-Being

Healthy emotion regulation is crucial for psychological functioning and may be one variable that can also help to protect health and, indirectly, to promote self-efficacy [16]. Historically, emotional psychological interventions have been focused on regulating negative and unpleasant emotions such as depressive and anxious symptoms. However, health and positive psychology have promoted an alternative approach that places positive emotions as the axis of change in these interventions [17]. Related to that, some studies have pointed to an association between positive well-being (i.e., positive affect) and a lower risk of a CVD event [18,19]. In particular, positive well-being has been found to be associated with lower odds of stroke [20], myocardial infarction, and the reduced probability of the recurrence of CVD [21,22]. Positive affect is also related to other CVD characteristics, such as biological responses that may be health protective, lower blood pressure, a lower level of cortisol, and less physiological activation [23,24,25]. Other studies indicate that the physiological reactivity to positive emotions acts as a counterbalance to the harmful reactivity of negative emotions, for example, helping patients to overcome the psychological consequences, including unpleasant emotions, after a CVD event [26,27]. From this positive perspective, a relationship is established between emotional well-being, focusing on positive emotions, and improving the emotional state of patients with CVD with better development and management of CVD [28].

Given the importance of experiencing positive emotions, having greater positive well-being, and the common anxiety-depression comorbidity [29], emotion regulation intervention oriented to CVD patients becomes highly recommended. Gross defined emotion regulation as “the processes by which individuals influence the emotions they have, when they have them and how they experience and express these emotions” [29], which includes the process of the identification, recognition, acceptance, and normalization of emotions. Healthy emotion regulation becomes critical to coping with challenging situations [30] such as a cardiac event. Even though there is not a lot of research that analyzes the relationship between emotion regulation and CVD, its influence on how patients with CVD deal with their disease seems clear. On the one hand, research has shown that patients with CVD had lower emotion regulation, which can, in some way, be negative to the prognosis of this chronic disease [16,31]. The use of unhealthy emotion regulation strategies may be responsible for the appearance of cardiovascular risk factors, such as body mass index, unhealthy diet, heavy alcohol consumption, sedentariness, etc. [16,32]. On the other hand, people diagnosed with CVD could be prone to deficits in emotion regulation [31]; hence, they would become more vulnerable to developing a mood disorder [33]. Then, for this bidirectional risk between emotional dysregulation and CVD risk factors, a psychological intervention to promote healthy emotional regulation seems necessary to decrease the odds of developing comorbid problems and, consequently, improve the quality of life of CVD patients.

### 1.3. mHealth

The term mHealth (mobile health) refers to the use of mobile devices, tablets, health-related applications, and other wireless technologies in health services, medical care, and clinical practice [34]. The incorporation of mHealth strategies aims to facilitate prescribing, adherence, patient communication, and health outcomes [34,35]. Currently, it has stimulated the use of mHealth tools, especially for risk groups such as CVD patients that may have some difficulties attending regular hospital follow-ups [36]. The unstoppable growth of the use of new technologies by the adult population favors the insertion of new techniques to promote, prevent, and intervene in health. The low adherence to treatment for CVD [37], which is probably due to its chronic nature, raises the need for interventions within the reach of these patients [38]. The evidence for this type of intervention is ambiguous so far. On the one hand, there is research that shows poor evidence of the effect of mHealth interventions to improve adherence to treatment (management and medication) in patients with CVD [39,40]. On the other hand, there are studies that point out the potential of mHealth to improve the adherence and management of chronic diseases [41] such as CVD [42]. This kind of intervention has also shown improvements in the physical and mental well-being of patients [43], favoring the management of depressive and anxiety symptoms [44].

### 1.4. The Present Study

The evidence reviewed above supports the relevance of incorporating an emotional psychological perspective to intervene with CVD patients. It also highlights the cost-effectiveness of brief psychological interventions, together with the promising results of the incorporation of mHealth strategies into healthcare. However, the literature addressing CVD interventions combining these components remains scarce. Therefore, the main aim of this study was to evaluate the effectiveness of a brief mHealth-based psychological intervention in emotion regulation to improve positive subjective well-being (enhancing positive affect) as well as self-efficacy for managing the disease in patients diagnosed with CVD. The first hypothesis was that participants included in the experimental group would have significantly greater positive subjective well-being and better self-efficacy for managing their chronic/cardiac disease compared to the control group. A secondary objective was to test if the expected differences between the groups would be maintained over time. Therefore, the second hypothesis was that better outcomes in positive subjective well-being and in self-efficacy for managing their chronic/cardiac disease in the experimental group would also appear in the follow-up evaluations.

## 2. Methods

### 2.1. Study Design

This was an interventional study, specifically, a two-arm non-randomized controlled prospective trial. The experimental group received a psychoeducational session in emotion regulation and a subsequent brief mHealth-based psychological intervention in emotion regulation, while the control group continued with their treatment as usual. The study was approved by the Andalusian Health Service’s Research Ethics Committee and the Reina Sofía Hospital in June 2015 (Acta 242, Ref. 2886, 29 June 2015).

### 2.2. Participants

Participants diagnosed with any type of CVD (angina pectoris, myocardial infarction, heart failure, arrhythmia, etc.) were recruited between March 2019 and April 2019 from the Cardiology Unit of Reina Sofía University Hospital of Córdoba, Spain. The inclusion criteria were: (1) women and men with a diagnosis of a CVD aged > 18, (2) ability to be fluent in Spanish, (3) having a smartphone compatible with the app used for the mHealth intervention (*WhatsApp*) and daily access to the internet, (4) having the required digital skills to follow the mHealth intervention, (5) not currently participating in another clinical trial, and (6) not currently receiving other psychological treatment. The exclusion criteria were: (1) women and men with a diagnosis of a CVD < 18 years, (2) not fluent in Spanish, (3) not having a smartphone compatible with the app used for the mHealth intervention and daily access to the internet, (4) not having the required digital skills, (5) serious mental health condition, (6) currently participating in another clinical trial, and (7) currently receiving other psychological treatment.

Potential participants (*N* = 132) were approached by telephone by an assistant researcher. Sixty-nine patients (*M* = 63.70 years, *SD* = 11.50) met the inclusion criteria and agreed to participate in the study, giving their informed consent. The participants were assigned to either the experimental group (*n* = 34) or the control group (*n* = 35) depending on their availability to attend the face-to-face session. There were seven dropouts and one death. Finally, 61 patients remained and completed all the study phases. The sociodemographic characteristics of the sample are described in Table 1.

### 2.3. Procedure

#### 2.3.1. Experimental Group

The experimental condition included a one-and-only face-to-face emotion regulation psychoeducational session and a subsequent mHealth-based emotion regulation psychological intervention.

*Psychoeducational session.* It was performed by a General Health psychologist in a private room at the Clinical Research Building of the Maimonides Biomedical Research Institute of Cordoba in small groups of about two to four people and lasted 60 min. The aim of this face-to-face session was psychoeducation about emotions, including identification, recognition, acceptance, and regulation, in order to facilitate the following mHealth intervention. Therefore, the session was structured in accordance with the above-mentioned objectives following the next headings and content: (a) What are emotions? Description and explanation of emotions concept, (b) Differences between basic and complex emotions: Provision of information about the different types of emotions regarding its nature, (c) Function and structure of emotions: Analyzing the function of emotions on a daily basis and, (d) Emotion regulation: Psychoeducation about the emotion regulation strategies, more specifically related to the management of their CVD diagnosis and provision of resources to improve emotion regulation.

In this session, two evaluations were conducted: the pre-test evaluation (baseline measurements) and after the psychoeducational session (post-session). In order to promote the intervention adherence, the patients were given a description of the mHealth intervention procedure with some motivational messages reinforcing their participation at the end of the session.

*mHealth intervention*. It started the day after the face-to-face session. The patients received for the next 14 days a *WhatsApp* message every day at the same time with an emotion regulation activity they had to perform (Supplementary File S1). The program of activities was based on Leahy et al. [45]. The order and content of the messages followed the scheme explained in the face-to-face session: the identification, recognition, acceptance, and regulation of emotions. The messages were prepared to be as brief and understandable as possible to be in accordance with brief psychological interventions.

After this intervention, the effectiveness of the mHealth intervention was evaluated (post-mHealth). To assess the changes, if any were maintained over time, two follow-up evaluations were included 2 weeks (follow-up 1) and 4 weeks (follow-up 2) after the mHealth intervention.

#### 2.3.2. Control Group

The participants of this group continued their regular medical follow-up without attending the psychoeducational session or receiving the mHealth intervention.

**Figure 1:**
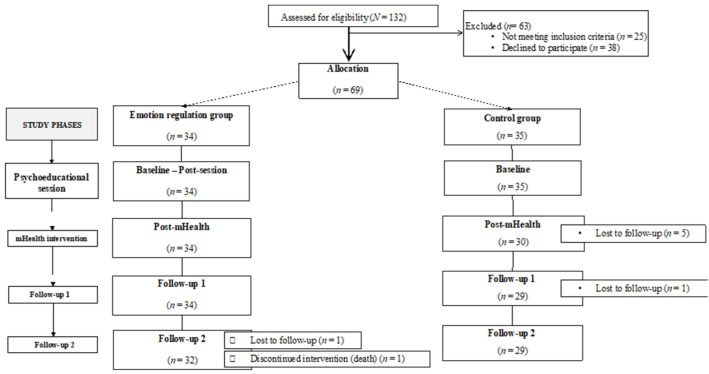
Flow chart of the sample and phases of the study.

Thus, each participant of the experimental group was assessed at five different time points (baseline, post-session, post-mHealth, follow-up 1, and follow-up 2), whilst the participants of the control group were evaluated four times (baseline, post-mHealth, follow-up 1, and follow-up 2, Figure 1). The baseline and post-session evaluations of the experimental group were measured in situ through an online questionnaire. The baseline measurements of the control group, as well as the three post-evaluations (post-mHealth, follow-up 1, and follow-up 2) of both groups were conducted by phone calls.

### 2.4. Outcome Measures

#### 2.4.1. Participant Characteristics

Sociodemographic characteristics including, age, sex, marital status, employment status, educational level, as well as the type of CVD and the level of limitation on activities of daily living (ADL) were asked of the participants. In addition, relevant psychological variables to the aim of the intervention, anxiety, and depression states and positivity, were also assessed to complete the description of the study sample. The scales used were the Spanish-validated version of the Hospital Anxiety and Depression Scale—HADS [46,47] (e.g., anxiety: Worrying thoughts go through my mind; depression: I still enjoy the things I used to enjoy)—and the Spanish-validated version of the Positivity Scale—P-scale [48] (e.g., I have great faith in the future).

#### 2.4.2. Primary Outcome

##### Positive Subjective Well-Being (PSWB)

It was assessed through the Positive Affect subscale (PA), from PANAS [49], the brief Spanish version [50]. The PA is a 10-item Likert-type scale that assesses to what extent participants experience pleasant emotions (e.g., To what extent do you feel interest [enthusiasm, inspiration]). The items are rated from 1 (not at all) to 5 (very much). The value for Cronbach’s alpha in the original study was 0.88 for this subscale. In this sample, the reliability value was α = 0.88 too.

#### 2.4.3. Secondary Outcome

*Self-Efficacy for Managing the Disease*. To provide a more complete evaluation of this outcome, it was assessed by means of two instruments that measure different types of disease management self-efficacy:

*Self-Efficacy for Managing Chronic Disease* (SEMCD—Spanish validated version) [51]. This Likert-type scale assesses self-efficacy for managing a chronic disease. It is composed of six items (e.g., How confident do you feel that you can keep the emotional distress caused by your disease from interfering with the things you want to do?) rated from 1 = (not at all confident) to 10 (totally confident). In the original study, Cronbach’s alpha coefficient was 0.85, and in this study sample, it was α = 0.86.

*Cardiovascular Management Self-Efficacy Scale* (CMSES—Spanish translated version) [52]. The CMSES was used to evaluate the perceived self-efficacy to manage the CVD. The CMSES is composed of nine items divided into three factors: cardiac risk (e.g., How well can you avoid problems or difficult situations and reduce sources of stress?), adherence to treatment (e.g., How well can you follow the prescriptions about food, even when you feel very nervous), and the recognition of cardiac symptomatology (e.g., How well can you recognize illness symptoms, such as palpitations, tachycardia, and short breath?). It uses a Likert-type scale of 5 points, from 1 (not at all confident) to 5 (totally confident). The reliability in the original study was α = 0.68; in this sample, it was 0.71.

In addition to the instruments mentioned above, a specific scale was created to obtain a subjective evaluation of the intervention at each phase of the study from the participants of the experimental group. This was a Likert-type scale composed of three items regarding adherence to the intervention, satisfaction with the intervention, and a general evaluation. The adherence to the intervention was measured in terms of frequency in reading and following the instructions of the mHealth intervention, from 1 (never) to 5 (every day). The degree of satisfaction with the messages received was evaluated from 1 (not at all satisfied) to 5 (completely satisfied), and finally, the general evaluation of the study was assessed from 1 (not at all satisfied) to 5 (completely satisfied). Cronbach’s alpha value for this scale was 0.75.

### 2.5. Data Analysis

The sample size was calculated using the G*Power 3.1.9.6 program [53] based on a previous study [54]. This study required a minimum of 34 participants in total to maintain a significance level of 0.05, an effect size of 0.25, and a power of 80.0%. The whole sample comprised 69 participants considering a dropout rate higher than 20% [55].

Student’s *t*-test, the chi-square test, and Fisher’s exact test were performed to compare the sociodemographic and clinical characteristics between groups. A dependent sample *t*-test was conducted to test if there were differences before and after the psychoeducational session for the experimental group. To test the effect of the intervention, a repeated-measures ANOVA was performed with each study variable with Time as a within-subject factor (baseline, post-mHealth, follow-up 1, and follow-up 2) and the experimental condition as a between-subject factor (experimental vs. control group). Bonferroni correction was used for pairwise comparisons. The data were analyzed using SPSS statistic software (v. 28).

## 3. Results

Table 1 shows the sociodemographic and clinical characteristics of the study sample. Statistically significant differences between groups were only found for employment status (*p* = 0.004). The baseline scores for each group on all outcome measures are shown in Table 2. No differences were found between the groups.

**Table 1:** Participant sociodemographic and clinical characteristics of the study sample.

	Total (*N* = 69)	Experimental Group (*n* = 34)	Control Group (*n* = 35)	Statistical Significance
Age (*M, SD*)	63.7 (11.5)	61.24 (11.1)	66.1 (11.6)	*t*(67) = 1.77, *p* = 0.081 ^a^
Sex, *n* (%)				*χ^2^*(1) = 1.95, *p* = 0.163 ^b^
Male	54 (78.3%)	29	25	
Female	15 (21.7%)	5	10	
Marital status, *n* (%)				*p* = 0.924 ^c^
Single	2 (2.9%)	1	1	
Single with partner	1 (1.4%)	1	0	
Married	57 (82.6%)	28	29	
Separated	2 (2.9%)	1	1	
Divorced	3 (4.3%)	2	1	
Widowed	4 (5.8%)	1	3	
Employment status, *n* (%)				*p* = 0.004 ^c^
Retired	40 (58%)	13	27	
Full-time work	21 (30.4%)	15	6	
Unemployed	6 (8.7%)	5	1	
Home care	2 (2.9%)	1	1	
Educational level, *n* (%)				*p* = 0.119 ^c^
Basic primary school	54 (78.3%)	24	30	
High school or higher	15 (21.7%)	10	5	
Type of CVD, *n* (%)				*p* = 0.677 ^c^
Angina pectoris	8 (11.6%)	3	5	
Myocardial infarction	33 (47.8%)	17	16	
Heart failure	5 (7.3%)	1	4	
Arrhythmia	5 (7.3%)	2	3	
Other	11 (15.9%)	7	4	
More than one of the above	7 (10.1)	4	3	
Level of limitation of ADLs, *n* (%)				
Level 1	29 (42%)	17	12	
Level 2	22 (31.9%)	10	12	
Level 3	14 (20.3%)	4	10	
Level 4	4 (5.8%)	3	1	
HADS (*M, SD*)	1.84 (0.49)	1.95 (0.44)	1.73 (0.52)	*t*(65) = −1.88, *p* = 0.065 ^a^
P-scale (*M, SD*)	3.94 (0.82)	3.89 (0.69)	3.99 (0.93)	*t*(67) = 0.50, *p* = 0.614 ^a^

Note. *M* = mean, *SD* = standard deviation, ADLs = activities of daily living, CVD = cardiovascular disease, HADS = Hospital Anxiety and Depression Scale, P-scale = Positivity scale. ^a^ Student’s *t*-test, ^b^ Chi-square test, ^c^ Fisher’s exact Test.

### 3.1. Psychoeducational Session

Table 3 shows the means, standard deviations, and *t*-test results of PSWB, SEMCD, and CMSES at the baseline and after the face-to-face session for the intervention group. The results from dependent *t*-test analysis showed differences between these two phases in positive subjective well-being and self-efficacy for managing the CVD, with higher scores in both scales in the post-session evaluation compared to the baseline.

### 3.2. mHealth Intervention

**Figure 2:**
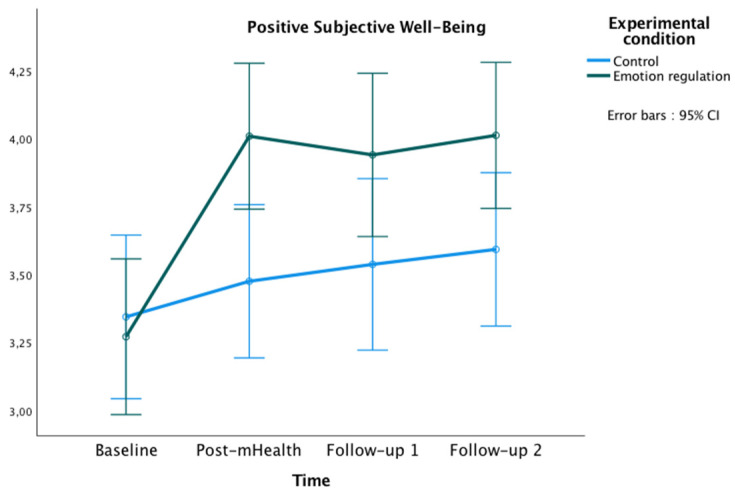
Changes in Positive Subjective Well-being in both groups over time.

**Figure 3:**
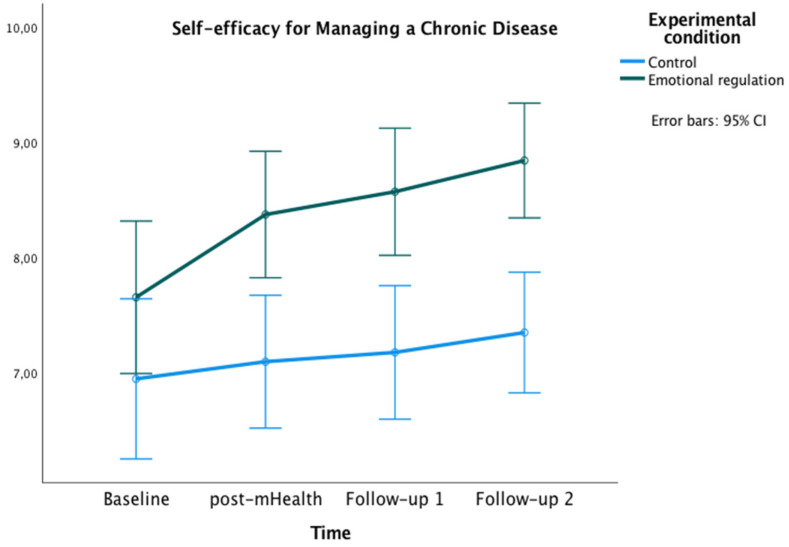
Changes in Self-efficacy for Managing Chronic Disease in both groups over time.

**Figure 4:**
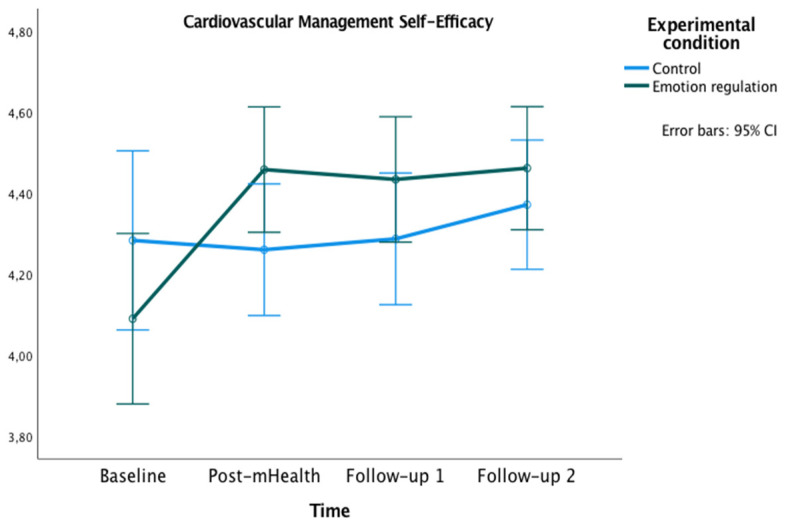
Changes in Cardiac Management Self-Efficacy in both groups over time.

The graphics presented in Figure 2, Figure 3 and Figure 4 show the marginal estimated means for both groups at the baseline and at the three post-test measures on PSWB, CMSES, and SEMCD.

#### 3.2.1. Primary Outcome

Positive Subjective Well-being (PSWB)

The repeated-measures ANOVA showed a significant main effect of time [*F*(3177) = 13.60, *p* < 0.001, ηp² = 0.19, observed power (OP) = 1.00], and a significant interaction effect of time x experimental condition [*F*(3177) = 4.70, *p* = 0.003, ηp² = 0.07, OP = 0.89]. Bonferroni pairwise comparisons showed significant differences between groups at post-mHealth (*M*_experimental_ = 4.01, *M*_control_ = 3.48, *p* = 0.008; 95% IC = [0.14, −0.92]), and follow-up 2 (*M*_experimental_ = 4.01, *M*_control_ = 3.59, *p* = 0.035; 95% IC = [0.03, 0.81]). Additionally, within the experimental group, some differences were found in PSWB between the study phases, being the scores higher in all post-evaluations compared to the baseline (all *p*s < 0.001).

#### 3.2.2. Secondary Outcome

##### Self-Efficacy for Managing the Disease

With regard to the SEMCD, a significant main effect of time was found [*F*(2,585,152.50) = 7.27, *p* < 0.01, ηp² = 0.11, potency = 0.97, Greenhouse-Geisser correction applied because Mauchly’s W = 0.759, *p* = 0.007]. Additionally, a main effect of the experimental condition was found [*F*(1) = 12.04, *p =* 0.001, ηp² = 0.17, OP = 0.93]. Bonferroni pairwise comparisons showed significant differences between groups at post-mHealth (*M*_experimental_ = 8.38, *M*_control_ = 7.10, *p* = 0.002; 95% IC = [0.48, 2.07]), follow-up 1 (*M*_experimental_ = 8.57, *M*_control_ = 7.19, *p* = 0.001; 95% IC = [0.60, 2.20]), and follow-up 2 (*M*_experimental_ = 8.84, *M*_control_ = 7.35, *p* < 0.001; 95% IC = [0.77, 2.22]). Within the experimental group, differences were also found with higher scores on SEMCD at all post-evaluations compared to the baseline (all *p*s < 0.05).

Related to the CMSES, a significant main effect of time [*F*(2,45,144.74) = 6.40, *p <* 0.001, ηp² = 0.10, OP = 0.94] and an interaction effect of time x experimental condition [*F*(2,45, 144.74) = 4.91, *p* = 0.005, ηp² = 0.08, OP = 0.86] were found. In both cases, the Greenhouse–Geisser correction was applied because Mauchly’s W = 0.70, *p* = 0.001. Bonferroni pairwise comparisons were not significant. However, the experimental group showed higher values on CMSES at post-mHealth (*M* = 4.46), follow-up 1 (*M* = 4.43) and follow-up 2 (*M* = 4.46) compared to the baseline (*M* = 4.09), all *p*s = 0.001.

### 3.3. Subjective Evaluation of the Intervention

The results indicated a great commitment and a positive evaluation of the intervention, showing differences over the three time point evaluations [*F*(2,62) = 15.5, *p* < 0.001, ηp²= 0.33, OP = 0.99]. Specifically, the results showed improvements in each phase, comparing post-mHealth (*M* = 4.27), follow-up 1 (*M* = 4.44), and follow-up 2 (*M* = 4.69), all *p*s < 0.05.

## 4. Discussion

The aim of this study was to evaluate the effectiveness of an mHealth-based brief psychological intervention in emotion regulation to enhance positive subjective well-being and self-efficacy to manage the chronic cardiac disease in patients with CVD. The study sample of 69 CVD patients (54 men, 15 women) was assigned to either the experimental (*n* = 34) or the control group (*n* = 35). Both groups were composed mostly of men in line with the sex distribution in CVD patients [56]. A face-to-face psychoeducational session followed by an mHealth intervention was conducted. Regarding the effectiveness of the face-to-face session, the results showed remarkable differences. The 1 h psychological intervention in emotion regulation strongly improved the patients’ positive subjective well-being. Moreover, this face-to-face session seemed to increase their perception of self-efficacy in managing the CVD. The mHealth emotion regulation intervention improved the positive subjective well-being of patients, as well as provided better management of the disease compared to the control group. According to the hypotheses of the study, the results showed a higher positive subjective well-being across the evaluations and a better cardiac and chronic management self-efficacy comparing both follow-ups with the baseline. Important differences over time were also found between the groups. The experimental group showed a greater positive subjective well-being in post-mHealth and follow-up 2, and a higher self-efficacy for managing chronic disease in post-mHealth, follow-up 1, and follow-up 2 compared to the control group.

These results corroborate those obtained by other studies, backing up the effectiveness of emotion regulation in improving psychological well-being [57,58,59]. The present results further suggest the effectiveness of psychological interventions in improving well-being in patients with CVD, according to previous research findings [28], as well as in enhancing self-efficacy in patients with CVD. These results are in line with several studies that analyzed the connection between positive affect, psychological well-being, and the management of CVD. Some of them found that patients with higher subjective well-being were prone to have healthier habits, such as taking care of diet, better sleep quality, reduced alcohol consumption, and better adherence to treatment [22,60,61,62], resulting in lower CVD risk [20,22].

Results from this study are consistent with research findings that highlight the importance of implementing emotion regulation techniques with patients who manifest cardiovascular problems [16,57,63] in order to favor healthy physical and psychological functioning, as well as reduce cardiovascular risk. Moreover, according to this study, an mHealth-based brief psychological intervention seems to be at least a good start to achieve these benefits in this population. This would not only be because of the increased subjective positive well-being, which would have already led to an improvement in quality of life and other health-related variables [64] but also because it has an effect, at least at the middle term, on the management of cardiac and chronic disease. Therefore, these findings contribute to the growing evidence that regards psychological well-being as a “bulwark” of health [19,20,59]. The significance and maintenance of the intervention effect in both self-efficacy measures and subjective well-being support the existing evidence of the lasting benefits of this brief type of educational intervention [10,11,12], giving strength to the idea of incorporating this kind of psychological intervention in cardiac rehabilitation programs [8,9].

In addition, as other studies have suggested, the use of new technologies allowed us to reach directly to the *patient’s hand*, which may have been one of the reasons for the high adherence to the intervention as shown in the results about the subjective evaluation in the study [41,42]. The effectiveness of a positive brief mHealth psychological intervention could imply a better adaptation to the disease, adherence to treatment, and the adoption of a new healthy lifestyle [8,60,61]. This may suppose an improvement in well-being and quality of life [28], reducing long-term risk factors such as comorbidities [5,6]. The results of this study seem to indicate that the combination of brief psychological interventions, due to their low cost and promptness, together with the adaptation of the treatments to the rising technological reality, are an attractive and effective alternative which can be considered from different approaches of health care when treating these patients [65].

### 4.1. Limitations

Although the results indicated the effectiveness of the intervention, this study has some limitations. The non-random allocation of the participants on the experimental conditions may bias the results. However, no differences between groups were found in clinical characteristics such as anxiety and depression states, positivity, or any outcome measure at baseline. Regarding the study sample size, though limited, it was similar to previous intervention studies with CVD patients [66,67]. Similar results are expected in wider samples, but this needs to be tested in future research. A potential bias in the data collection also needs to be mentioned. Baseline measurements were conducted differently for the experimental and the control groups (in situ vs. phone call, respectively), which could have affected the study results. However, as mentioned before, no differences between the groups were found at baseline, indicating that the different procedure would have not affected the participants’ responses. Finally, the low presence of women in the study sample is consistent with the pattern of a higher prevalence of most CVD in men. However, several studies argue that this sex prevalence ratio is based on a gender bias in the diagnosis of CVD [56]. Relating to that, in our study, the underrepresentation of women is higher in the experimental group (29 men/5 women) compared to the control group (25 men/10 women). This lower enrolment of women in CVD programs has been observed in other studies [68]. This could be explained, among other things, by the underestimation of CVD risk in women, a lower importance of self-care, as well as some gender barriers (e.g., lower social and family support, transport-economic problems, lack of time due to the caregiving role) [69]. Although this issue might bias the results, it is important to note that in our study there were no statistical differences in the sex representation between groups.

### 4.2. Future Research

Regarding future lines of research, it would be interesting to evaluate and compare the effectiveness of the intervention proposed with other types of intervention modalities (i.e., only face-to-face, only mHealth, and no treatment). This would shed light on the effect of interventions mediated by the incorporation of mHealth strategies. It is also proposed to go one step further and take advantage of the fact that mHealth interventions are projected as a patient-centered strategy to encourage a personalized intervention, a tailored communication, considering the individualities of the patients with regard to the objective of the intervention. In this line, the gender bias mentioned above should be taken into account when designing future interventions in CVD. The personalization of these interventions would allow to address the specific characteristics of women, facilitating their enrolment and hopefully improving their CVD-related outcomes. In addition, other variables could also be evaluated at baseline, considering the gender perspective and the profiles of patients with CVD. Furthermore, the possibility of adding biomarkers in future research should be contemplated in order to obtain richer and likely less-biased information on the intervention.

## 5. Conclusions

This study shows the effectiveness of an mHealth-based brief psychological intervention in emotion regulation to enhance positive subjective well-being (showing an increase in positive affect) and to improve self-efficacy in the management of chronic and cardiac disease in CVD patients. The adaptation of psychological interventions with new technologies and new forms of understanding life and healthcare treatments was a good option to reach different patient profiles and to promote adherence to the psychological intervention. The results of this study are significant as they provide evidence on how brief psychological interventions together with mHealth are a good combination treatment for CVD patients. It has been proved that with a low cost and promising benefits, together they can contribute to an improvement in psychological well-being and the management of the disease that may translate in the long term to a better quality of life of CVD patients.

## Figures and Tables

**Table 2:** Baseline scores in the experimental and the control group in all the outcome measures.

	Experimental Group (*n* = 34)		Control Group (*n* = 35)		
	M	SD	M	SD	p
PSWB	3.28	0.76	3.36	0.85	0.674
SEMCD	7.71	1.79	6.85	1.92	0.059
CMSES	4.11	0.66	4.31	0.47	0.144

Note. *M* = mean, *SD* = standard deviation, PSWB = Positive Subjective Well-Being, SEMCD = Self-Efficacy for Managing Chronic Disease, CMSES = Cardiovascular Management Self-Efficacy Scale.

**Table 3:** Baseline and post-session scores of PSWB, SEMCD, and CMSES in the experimental group.

	Baseline		Post-Session (Face-to-Face)		Baseline–Post-Session Emotion Regulation		
	*M*	*SD*	*M*	*SD*	*t*(33)	*p*	*d*
PSWB	3.28	0.76	3.93	0.68	−6.60	<0.001	0.57
SEMCD	7.71	1.79	8.02	2.00	−1.40	0.170	1.31
CMSES	4.11	0.66	4.26	0.67	−2.42	0.021	0.35

Note. *M* = mean, *SD* = standard deviation, PSWB = Positive Subjective Well-Being, SEMCD = Self-Efficacy for Managing Chronic Disease, CMSES = Cardiovascular Management Self-Efficacy Scale.

## Data Availability

The data presented in this study are available on request from the corresponding author.

## Supplementary Materials

The following supporting information can be downloaded at: https://www.mdpi.com/article/10.3390/healthcare10091640/s1, Supplementary File S1: WhatsApp Messages from the mHealth Intervention.
