# Supplementary material for: A Brief mHealth-Based Psychological Intervention in Emotion Regulation to Promote Positive Subjective Well-Being in Cardiovascular Disease Patients: A Non-Randomized Controlled Trial
Source: Healthcare (Basel). 2022 Aug 28;10(9):1640. doi: 10.3390/healthcare10091640 (PMC9498759; doi:10.3390/healthcare10091640)
Supplement: Supplementary file 1 [file healthcare-10-01640-s001.zip › healthcare-1866240-supplementary.pdf]

healthcare

MDPI

Supplementary File S1 WhatsApp Messages from the mHealth Intervention

1. Good morning! Identifying our emotions

Today, we propose to you an exercise to help you become aware of your emotions. Stop for a moment and reflect on how you feel today. Collect the emotion or emotions you are feeling at this moment.

2. Good morning! The pleasant emotions

Today, we suggest you pay special attention to those emotions that please you (joy, satisfaction, peacefulness, pleasure, love, hope, confidence, etc.). When we look at pleasant emotions, we realize that we experience them more than we think!

3. Good morning! Recognizing emotions

Today, we ask you to think of at least one emotion you felt yesterday or today and write down:

What happened right before you felt this emotion?

What did you feel (physical sensations)?

What is the name of this emotion?

How did you express this emotion?

4. Good morning! Emotion or thought?

Emotions are often accompanied by thoughts, and it is easy to confuse them. Today, we suggest that you remember one emotion you felt yesterday and write down what you thought when you felt that emotion.

5. Good morning! What are emotions for?

All emotions have a function. For example, fear protects us from possible danger. Remember some unpleasant emotion you have experienced recently and think about what that emotion did for you.

6. Good morning! Emotions are temporary

Some emotions can last all day, while others only last a few minutes. Today, we propose that you write down at least one emotion you are feeling right now and score it from 0 (nothing) to 10 (completely) according to the intensity of the feeling. At the end of the day, score the same emotion again. You will probably find out that the intensity of the emotion has changed.

Knowing that emotions are temporary helps us to regulate them.

7. Good morning! Transforming emotions

Sometimes, we feel unpleasant emotions, and we don't know how to get rid of them. It can be helpful to remember a happy moment, for example, the birth of a child, a family celebration, an important achievement, etc. and take time to remember how you felt at that moment. This will help you to change the unpleasant emotion into a pleasant one.

8. Good morning! Regulating unpleasant emotions

Take some time to remember a difficult moment in your life that emotionally overwhelmed you, what did you do to get through the situation? Did your way of handling emotions at that time help you? Would you do the same now?

9. Good morning! Music to regulate emotions

Today, we ask that you spend a few minutes listening to a song you like and observe how you feel while listening to it. Has your mood improved? Listening to music can be a good strategy to help us manage our emotions.

10. Good morning! What can we do when we feel unpleasant emotions?

There are several things we can do to manage emotions in a healthy manner. Today, we propose that you go for a 10–15 minute walk or do some activity that you like. Remember, physical exercise helps us to manage our emotions!

11. Good morning! Models of emotional management

Think of a person who manages his or her emotions properly. Try to apply what that person does to improve yourself. Learn from these models and remember those strategies for managing your emotions.

12. Good morning! Helping other people manage their emotions

Identify a difficult situation you have been through or are currently going through. Considering your experience, as well as what you have been working on with emotions these days, how would you advise someone close to you if they were going through the same situation?

13. Good morning!

Practicing what you have learned. Now that you are able to identify different, basic and complex emotions, express in detail how you feel today.

14. Good morning!

Thank you very much for participating in this study. This is the last message. For the next 14 days, we ask you to do this activity: whenever you feel an unpleasant emotion, give yourself time to experience and understand it, and then we encouraged you to do an activity that will make you feel better.
